# Development of an Open Microfluidic Platform for Oocyte One-Stop Vitrification with Cryotop Method

**DOI:** 10.3390/bios12090766

**Published:** 2022-09-19

**Authors:** Shu Miao, Chenxi Guo, Ze Jiang, Hao-Xiang Wei, Xin Jiang, Jingkai Gu, Zhuo Hai, Tianren Wang, Yun-Hui Liu

**Affiliations:** 1 School of Mechanical Engineering and Automation, Harbin Institute of Technology, Shenzhen 518055, China; 2 Shenzhen Key Laboratory of Fertility Regulation, Reproductive Medicine Center, The University of Hong Kong-Shenzhen Hospital, Shenzhen 518005, China; 3 The T Stone Robotics Institute, Department of Mechanical and Automation Engineering, The Chinese University of Hong Kong, Hong Kong SAR, China

**Keywords:** open microfluidic chip, cell manipulation, vitrification

## Abstract

Oocyte vitrification technology is widely used for assisted reproduction and fertility preservation, which requires precise washing sequences and timings of cryoprotectant agents (CPAs) treatment to relieve the osmotic shock to cells. The gold standard Cryotop method is extensively used in oocyte vitrification and is currently the most commonly used method in reproductive centers. However, the Cryotop method requires precise and complex manual manipulation by an embryologist, whose proficiency directly determines the effect of vitrification. Therefore, in this study, an automatic microfluidic system consisting of a novel open microfluidic chip and a set of automatic devices was established as a standardized operating protocol to facilitate the conventional manual Cryotop method and minimize the osmotic shock applied to the oocyte. The proposed open microfluidic system could smoothly change the CPA concentration around the oocyte during vitrification pretreatment, and transferred the treated oocyte to the Cryotop with a tiny droplet. The system better conformed to the operating habits of embryologists, whereas the integration of commercialized Cryotop facilitates the subsequent freezing and thawing processes. With standardized operating procedures, our system provides consistent treatment effects for each operation, leading to comparable survival rate, mitochondrial membrane potential (MMP) and reactive oxygen species (ROS) level of oocytes to the manual Cryotop operations. The vitrification platform is the first reported microfluidic system integrating the function of cells transfer from the processing chip, which avoids the risk of cell loss or damage in a manual operation and ensures the sufficient cooling rate during liquid nitrogen (${\mathrm{LN}}_{2}$) freezing. Our study demonstrates significant potential of the automatic microfluidic approach to serve as a facile and universal solution for the vitrification of various precious cells.

## 1. Introduction

Oocyte cryopreservation is an effective technique widely used in human-assisted reproductive technology and fertility preservation [1]. Through collecting and preserving oocytes at a young age, the cryopreservation technique preserves the dramatically declined ability of oocytes to fertilize and develop with age and allows women to delay pregnancy until they wish to have a child [1,2,3]. Since Chen et al. reported the first successful cryopreservation of human oocytes in 1986 [4], considerable efforts have been paid to address the challenges of mature oocytes cryopreservation, including the large cell volume, small relative surface area, high water content, special intracellular contents and the presence of meiotic spindles [1,5,6,7,8].

**Figure 1:**
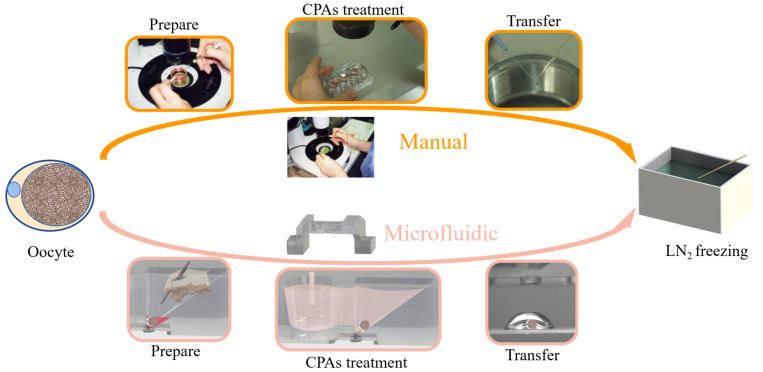
Schematic showing manual and microfluidic vitrification approaches. Vitrification involves multiple steps of cell pick-and-place before freezing in liquid nitrogen.

Oocyte cryopreservation technology can be categorized into slow-rate freezing and vitrification according to different freezing principles [9,10,11,12]. In a vitrification process, the cells are rapidly cooled down by direct or indirect contact with liquid nitrogen (${\mathrm{LN}}_{2}$), leading to a glass-like solidification of biological samples in the absence of ice crystals formation [13]. Currently, vitrification is the most commonly used method for the cryopreservation of human oocytes due to improved survival and pregnancy rates. The most commonly used vitrification method in clinical practice is the Cryotop method [5,14], as shown in Figure 1. This operation mode has the advantages of high freezing efficiency and convenient operation, and is accepted by most embryologists as the gold standard. During Cryotop method for vitrification, the washing sequences and timings for each CPA (including equilibrium solution (ES) and vitrification freezing solution (VS)) treatment should be precisely controlled to avoid the overexposure of cells to CPAs, which will cause irreversible osmotic damage to oocytes. The clinical operation of the Cryotop method is manually performed by skilled embryologists within 15–20 min; however, the failure of manual vitrification still occurs due to cell loss, mechanical and osmotic damage etc. [15]. It is necessary to develop a standardized protocol for oocyte vitrification based on the Cryotop method, which is more reliable and minimizes the influence of operators.

Microfluidic systems have been demonstrated as promising solutions for various applications in assisted reproduction, such as cumulus–oocyte complexes (COCs) removal, sperm screening, in vitro fertilization and embryo culture [16,17,18,19]. Through precisely manipulating the fluids at the microscale, microfluidic systems can realize complex fluid behaviors such as co-flow diffusion, droplet generation, and concentration gradient generation using different channel designs [20,21,22,23,24]. For cryopreservation of oocytes and embryos [6,25,26,27,28,29,30,31,32], two categories of microfluidic platforms—confinement devices and free-flow devices—have been developed to simplify the process of CPAs loading and unloading [33].

Confinement devices use various mechanisms (e.g., physical obstruction, pressure difference or electrowetting) to manipulate oocytes/embryos [6,25,26,27,28,29,30,32] and provide a temporal concentration gradient of CPAs around the cells to complete vitrification pretreatment. Guo et al. designed a curved-channel and microcolumn array microfluidic device to immobilize the oocyte and linearly load CPAs under diffusion, which reduced the permeability damage caused by sudden changes in CPAs concentration [6]. Tirgar et al. fabricated a microchannel with a width smaller than the embryo size to limit the movement of the embryo, and integrated a capillary pump to introduce the CPAs instead of the microsyringe pump, which simplified the operation process [28]. Pyne et al. developed the EWOD platform and used the digital microfluidic method to complete the vitrification pretreatment of embryos [26]. In a confinement device, the CPAs surrounding the oocyte can be controlled throughout the loading and unloading process through diffusion, enabling operators to perform experiments with concentration profiles, which can be hardly achieved with manual vitrification.

Nevertheless, the fine confinement structure causes considerable difficulties in chip fabrication and packaging. In contrast to confinement devices, free-flowing devices do not immobilize cells, and instead construct multiple parallel channels with adjacent channels and control the environment of CPAs around cells through diffusion under laminar flow [31]. These devices introduce oocytes/embryos and CPAs into parallel channels and allow CPAs to freely diffuse into cells through long serpentine channels. Despite the simpler fabrication process and more stable concentration change compared with confinement devices, it is difficult to correct the position of the oocyte/embryo in the channel of free-flowing devices and the operation period is much longer. It should be noted that most of the existing confinement and free-flow devices are closed systems, which means that the oocyte/embryo will be put into ${\mathrm{LN}}_{2}$, accompanying the bulky chips. As a result, the cooling rate of the cells will be dramatically decreased and may not reach the requirement for vitrification. Therefore, it is critical to construct a microfluidic platform that enables transfer of the cell with droplet volume to Cryotop accurately with sufficiently high cooling rate.

Herein, we report a convenient, universal, user-friendly and high-efficiency microfluidic system for oocytes vitrification, which consists of an open microfluidic chip and a companion device. The design of the open microfluidic chip is based on the Cryotop method, using a confinement structure without packaging and integrating the transfer channel and capillary valve, which reduces the difficulty of fabrication and operation. Compared to the closed systems, our open chip (similar to a Petri dish) is more convenient and more consistent with the operating habits of embryologists. This system provides a simplified and standardized vitrification process in which the operator needs only to load the oocyte into the designated area of the chip and operate the companion device to obtain the same vitrification pretreatment effect as the Cryotop method. Compared with manual operation, the open microfluidic system avoids the risk of cell loss or damage caused by frequent oocyte movement, the loading of CPAs in a concentration gradient reduces osmotic damage and the standardized process eliminates uncertainty of manual operation. In this study, mouse oocytes were selected as experimental candidates and vitrified through the manual and microfluidic protocols, which were then examined after a standard thawing process. The results showed no significant difference in survival rate and quality (characterized by MMP and ROS level) among thawed oocytes treated by the two methods, demonstrating the validity of our system.

In short, to achieve the platform for oocyte vitrification, the contributions of this research work can be summarized as follows: (1) a newly designed prototype based on the clinical vitrification Cryotop method, featuring an embryologist-centered configuration; (2) a novel open microfluidic chip based on convection–diffusion and surface tension, validated through numerical simulation and experiments; (3) evaluation of system reliability based on the survival rate, MMP and ROS level of mouse oocytes.

## 2. Materials and Methods

### 2.1. Device Design and Fabrication

**Figure 2:**
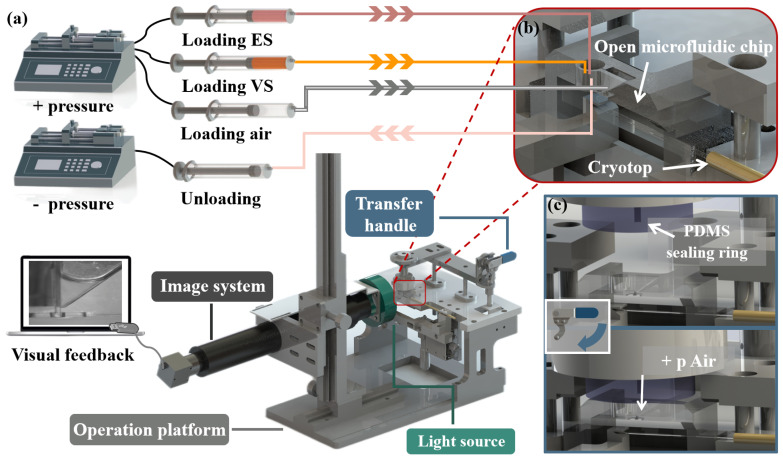
The structure diagram of the vitrification system and working principle of system. (**a**) The vitrification system composed of the open microfluidic chip and the companion system (syringe pumps, operation platform, transfer system and the imaging system). The operator manual placed the oocyte into the chip, completed the vitrification pretreatment and transferred with the assistance of the companion system. The real-time internal conditions of the chip on the whole operation process were presented to the operator by visual feedback. (**b**) Above all, Cryotop and the open microfluidic chip containing the oocyte were fixed on the operation platform. The syringes used for loading (ES, VS and air) and unloading were, respectively, connected to the chambers on both sides of the chip and PDMS sealing ring with soft silicone tubes. (**c**) Schematic diagram of the transfer process. After vitrification pretreatment, the operator pressed the transfer handle, and the PDMS sealing ring was pressed on the upper surface of the open microfluidic chip to form a hermetic space. The oocyte was transferred to Cryotop by external pressure.

**Figure 3:**
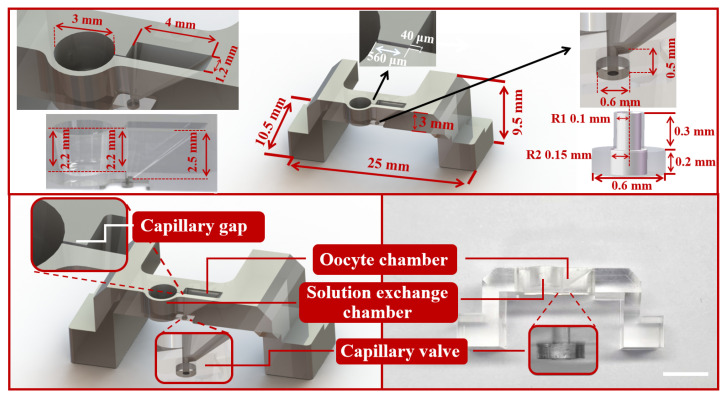
The dimension detail, structure diagram and physical diagram of the open microfluidic chip, composed of capillary gap, oocyte chamber, solution exchange chamber and capillary valve. Scale bars are 2 mm.

During oocyte vitrification, the main challenge is to ensure the quality of the CPAs exchange and the manipulation of the oocyte. The high-efficiency CPAs exchange directly affects the survival rate and development rate of oocytes. Compared with the traditional closed microfluidic, the open microfluidic is more user-friendly and has a simpler manufacturing process. In this work, an open microfluidic chip with the companion device was developed to achieve oocyte vitrification pretreatment and transfer the oocyte to the Cryotop after pretreatment (Figure 2). The open microfluidic chip (Figure 3) consisted of two independent chambers (left: solution exchange chamber, right: oocyte chamber), a capillary gap, a transfer channel and a capillary valve. The solution exchange chamber was a cylindrical shape with a radius of 1.5 mm and a depth of 2.2 mm, designed for the loading and unloading of CPAs. The oocyte chamber was a 2.5 mm depth square conical shape, with 4 mm (length) × 1.2 mm (width) on the upper surface and 0.4 mm (length) × 0.3 mm (width) on the bottom, designed for positioning and placing the oocyte. The capillary gap, having dimensions of 40 μm (width) × 560 μm (length) × 2.2 mm (depth), as a bridge, connected the chambers on both sides to achieve the solution exchange by spontaneous capillary flow (SCF) and prevent the oocyte (diameters in the range of 70 to 100 μm) from being lost in the process of treatment [34,35]. The oocyte chamber (2.5 mm) was deeper than the capillary gap (2.2 mm) and ensured that a certain volume of liquid would be contained around the oocyte to prevent osmotic damage caused by evaporation after the solution unloading. The transfer channel with a radius of 100 μm was designed to connect the oocyte chamber, and the capillary valve (upper part: radius 100 μm, depth 300 μm; bottom part: radius 150 μm, depth 200 μm). In microfluidic chips, the influence of gravity and inertia is reduced at the microscale, and the influence of surface tension becomes significant and even dominates under certain conditions [36]. A capillary valve was designed to temporarily block the transfer channel, in which the liquid was simply stopped by the surface tension caused by a sudden expansion of the microchannel cross-section. After vitrification pretreatment of the oocyte, the capillary valve was opened by the introduction of positive pressure.

The companion device consisted of two syringe pumps (LSP02, LONGER, Baoding, China), the operation platform, the transfer system (includes PDMS sealing ring and a transfer handle) and the imaging system (Figure 2a). One of the syringe pumps provided positive pressure (to load CPAs and transfer the oocyte) and the other provided negative pressure (to unload CPAs). During oocyte vitrification pretreatment, CPAs were loaded and unloaded by syringe pump. During the transferring process (Figure 2c), the operator pressed the transfer handle (PDMS sealing ring was pressed above the chip) and the syringe pump provided positive pressure for opening the capillary valve; then, the oocyte was transferred to the Cryotop. The imaging system, including a CCD camera (MER-503-36U3M, DAHENG IMAGING, Beijing, China) and a light source (CR-6390-R, DAHENG IMAGING, Beijing, China), was used to track the oocyte in the chip. Considering that the oocyte would suffer damage when exposed to a light source, we chose a red LED (Peak wavelength: 623nm), which could minimize the damage to the oocyte. The chip and the companion device were fabricated by acrylic and aluminum alloy, respectively, through computer numerical control (CNC) machining (SuZhou Wenhao Co, Ltd, Suzhou, China).

### 2.2. System Operation

The CPAs (ES and VS) were prepared in HEPES-buffered tissue culture medium (Invitrogen, Waltham, MA, USA) supplemented with 10%*v/v* fetal bovine serum (FBS; Invitrogen, Waltham, MA, USA) based on the typical protocols. Briefly, the ES contained 7.5%*v/v* dimethyl sulfoxide (DMSO; Sigma-Aldrich, Darmstadt, Germany) and 7.5%*v/v* ethylene glycol (EG; Sigma-Aldrich, Darmstadt, Germany), and the VS contained 15%*v/v* DMSO, 15% *v/v* EG and 0.4 mol/L sucrose (Sigma-Aldrich, Darmstadt, Germany).

**Figure 4:**
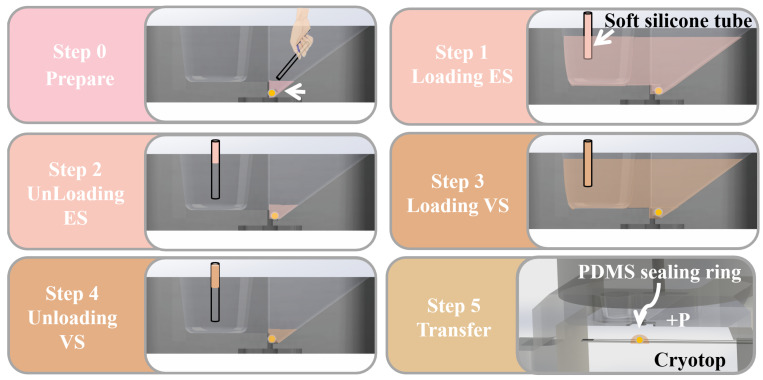
The operation steps of vitrification on the open microfluidic chip: preparation, loading and unloading ES/VS and transfer.

Above all, for the operator needed to prepare 4 disposable syringes (5mL range, BD, Franklin Lakes, NJ, USA), three of them (loading ES, VS and air, respectively) were placed in the syringe pump that provided positive pressure, and the other one (no loading) was placed in the syringe pump that provided negative pressure. The syringes (loading ES, VS and no loading) were connected with the solution exchange chamber of the open microfluidic chip with soft silicone tubes, while the loading and unloading of CPAs were controlled by the syringe pump, respectively. Finally, a soft silicone tube was used to connect the syringe (loading air) to the gas port on the PDMS sealing ring, and Cryotop was placed in the designated position. After the preparations were completed, the overall vitrification protocol (Figure 4) was divided into the following steps:

- Step 0: The operator manually places the oocyte at the bottom of the oocyte chamber with a small amount of medium through the stereo microscope.
- Step 1: Load 20 μL ES (50 μL/min) into the solution exchange chamber. Part of the ES will be introduced into the oocyte chamber through the spontaneous aspirating of the capillary gap. Wait for 9–12 min for equilibration of the oocyte in ES.
- Step 2: Unload ES (100 μL/min) from the solution exchange chamber until the liquid level of residual solution around oocyte is below the capillary gap.
- Step 3: Load 20 μL VS (50 μL/min) into the solution exchange chamber; part of the VS will be introduced into the oocyte chamber, in which the oocyte is treated for 30 s. Note that due to the high concentration of cryoprotectant in VS, excessive operation time will have adverse effects on the oocyte. Steps 3–5 should be completed within 1 min.
- Step 4: Unload VS (100 μL/min) from the solution exchange chamber until the liquid level of residual solution around oocyte is below the capillary gap, which is similar to Step 2.
- Step 5: After the CPAs exchange is completed, the operator presses the transfer handle to transfer the oocyte from the channel to the Cryotop by syringe pump. Finally, the operator puts the Cryotop into ${\mathrm{LN}}_{2}$ to complete the vitrification.

### 2.3. Fluid Simulation

The critical designs of our microfluidic chip are the variation rate of CPA concentration in the oocyte chamber and the reliability of the capillary valve. Therefore, to ensure that the designed chip can meet the requirements, numerical simulations of the fluid behavior in the abovementioned key structures were performed using COMSOL Multiphysics 5.6.

In the process of CPAs treatment, CPAs were introduced into the oocyte chamber through the capillary gap, and the CPAs concentration around the oocyte was changed by diffusion. The gradual change of CPAs concentration avoided the osmotic pressure shock caused by traditional manual operations. However, the cytotoxic solutes in the CPAs might cause damage to oocytes if the treatment time was too long due to the relatively slower diffusion process. To ensure that the concentration of CPAs around the oocyte could reach the concentration required for pretreatment, we used numerical simulation to predict the time-dependent changes in the concentration in the right chamber. Since the concentration of solute in CPAs was low compared with the solvent, it could be assumed that solute molecules only interact with water molecules in the solvent. Thus, the diffusion of CPAs concentration around oocytes could be described by Fick’s law:

(1) −∇·(−D∇c+cu)=0

where *c* represents the concentration and u is the fluid velocity field. In the process of CPAs processing, VS processing played a vital role because the time and concentration needed to be strictly controlled to prevent the damage to the oocyte. The solutes within VS were mainly EG, DMSO and sucrose, among which sucrose had the lowest concentration and diffusion coefficient. Therefore, we selected sucrose, as a candidate, and numerically simulated its transient concentration in the oocyte chamber.

**Figure 5:**
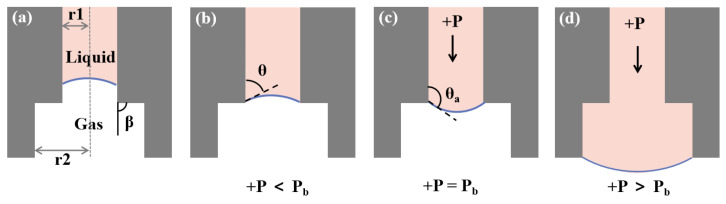
Different situations of liquid flow in capillary valve. (**a**) The internal structure diagram of the capillary valve; the inner radius (r1: 100 μm, r2: 150 μm) had two sudden expansions and the channel expansion angle β is 90${}^{\circ }$. (**b**) The liquid reached the capillary valve, without external pressure, and the meniscus stopped at the edge of the microchannel outlet. (**c**) The external pressure gradually increased, and the convexity of the meniscus and contact angle became larger. (**d**) When the external pressure exceeded the critical burst pressure, the capillary valve was opened and droplet was transferred to Cryotop.

Another critical part of our chip is the capillary valve, which was a passive valve and used surface tension to control fluid flow. When the external driving pressure was less than the critical value, the valve was closed. Once the external driving pressure exceeded the critical value, the valve would be opened and transfer the oocyte to the Cryotop. In order to ensure the reliability of the capillary valve, it was necessary to determine the critical burst value of the capillary valve. In the capillary valve we designed (Figure 5), there were two microchannel sections with sudden expansion (the first: the radius increases from 100 μm to 150 μm; the second: the radius 150 μm to the outside). When a liquid column was trapped in a vertical capillary of radius *R*, the capillary force should be equal to the downward force *F* acting on the liquid column to reach equilibrium [37,38]. The capillary force was generated by the meniscus on the lower surfaces of the liquid column. The external force *F* might originate from the pressure difference between the upper and lower surfaces and the gravity of the liquid column itself. Therefore, under ideal conditions:

(2) $$-2\pi R\gamma_{lg}\cos\theta =F$$

where $\gamma_{\mathrm{lg}}$ is the liquid–gas surface tension coefficient and θ represents the contact angle of the meniscus. In our chip (Figure 5a), the external force *F* consisted of the weight of the liquid column and the external pressure over the cross-sectional area. When the liquid reached the capillary valve, the meniscus stopped at the edge of the microchannel outlet (Figure 5b). Under the action of external driving pressure, the meniscus deformed until its contact angle with the wall reached the critical advancing angle value $\theta_{a}$ (Figure 5c). Then, the valve opened and the meniscus continued to move forward (Figure 5d). The analytical expression of burst pressure ($P_{b}$) could be obtained according to Equation (2):

(3) $$P_{b}=-2\gamma_{\mathrm{lg}}\frac{\cos\left(\theta_{a}+\beta \right)}{r}$$

where β is the channel expansion angle and the r is its radius. When $\theta_{a}$ + β is equal to π the burst pressure $P_{b}$ will reach its maximum value. The burst pressure was mainly determined by the surface tension coefficient and the channel radius. The radius at the second sudden expansion of the microchannel cross-section (150 μm) was greater than that at the first (100 μm); therefore, the burst pressure of the second was less than that of the first. The second structure was only used to protect the liquid column from contacting the outside as much as possible.

At the interface between the liquid phase and the gas phase, the level set method was used to describe the behavior between the two phases on the interface. In order to predict the burst pressure value, a numerical model was established to simulate the change of the meniscus shape under different driving pressures.

### 2.4. Characterization of the Open Microfluidic Performance

The concentration of CPAs in the oocyte chamber was experimentally characterized using fluorescent tracer. We used sodium fluorescein (Sigma-Aldrich, Darmstadt, Germany), having a molecular weight (MW: 332.3 g/mol) close to sucrose (MW: 342.3 g/mol), as a tracer. The fluorescence intensity of the culture medium containing different concentrations of tracer was quantified at 490 nm, leading to the correlation between tracer concentration and fluorescence intensity. Then, we added the culture medium (0.4 mol/L) to the solution exchange chamber and recorded the changes in the fluorescence intensity of the oocyte chamber in real time.

To obtain the burst pressure of the capillary valve, a constant pressure pump (PC01, Fluidiclab, Shanghai, China) was used to provide additional pressure to the interior of the oocyte chamber, which was prefilled with VS. The variation of the meniscus at the capillary valve was characterized under different pressures.

The performance of the chip to realize the CPAs exchange and oocyte transfer was evaluated by an imaging system. Since the real oocyte was almost transparent and hard to observe, opaque polystyrene (PS) microparticles (Rigor Science, Wuxi, China) with a similar diameter to oocytes (80 μm) were used to verify the effectiveness of the chip design. After the microparticle was placed into the oocyte chamber, the operator conducted the abovementioned vitrification procedures while the imaging system monitored the liquid level and the particle position inside the chip.

### 2.5. Mouse Maintenance and Oocytes Collection

Female C57BL/6J mice (The Jackson Laboratory, Bar Harbor, ME, USA) between 6 and 10 weeks of age were used in this study. The animals were housed under a controlled environment with free access to water and food, and with lights switched on between 6:00 and 18:00. All experimental protocols were approved by the regional ethics committee of the University of Hong Kong–Shenzhen Hospital. To collect fully grown germinal vesicle (GV) oocytes, mice were super-stimulated with the injection of 10 IU pregnant mare serum gonadotropin (PMSG; Cat. G4527, Sigma, St. Louis, MO, USA).The COCs were obtained by manually rupturing antral ovarian follicles from mice ovaries 48 h after injection of PMSG. Cumulus cells were removed by mechanically repeated pipetting via a 75 μm (in diameter) tip (Cat. MXL3-IND-75, The STRIPPER^®^ Tips, ORIGIO, CooperSurgical, Målov, Denmark) before collecting ready-to-use GV oocytes. To collect MII oocytes, mice were injected with 10 IU PMSG first; after 48 h, the mice were injected again with 10 IU hCG (Cat. HOR-250, Chorionic Gonadotropin Human, PROSPEC, Kenilworth, NJ, USA). The MII oocytes were obtained by punctuation of tubal enlargement after 16 h of hCG injection. The GV and MII oocytes were cultured in M2 medium (Cat. M7167, Sigma, St. Louis, MO, USA) at 37 ${}^{\circ }$C before the cryopreservation.

### 2.6. Manual Vitrification Procedure

Manual vitrification of the oocyte was accomplished using our previously configured ES and VS solution and Cryotop. Specifically, in the vitrification process, the operator used an embryo transfer tube to transfer the oocytes into ES and VS sequentially, and the processing time was 9–12 min and 30 s, respectively. The oocyte was placed on the Cryotop; then, the Cryotop was inserted into ${\mathrm{LN}}_{2}$ to complete vitrification.

### 2.7. Thawing Procedure

The oocytes vitrified by manual operation and microfluidic system were manually thawed using the Cryotop Kit (Kitazato BioPharma, Shizuoka, Japan). During the thawing process, the Cryotop with vitrified oocyte was removed from the ${\mathrm{LN}}_{2}$, and the tip of Cryotop was quickly immersed into a prewarmed (37 ${}^{\circ }$C) thawing solution (TS) for 1 min. Then, the oocyte was transferred into a dilution solution (DS), washing solution (WS1) and washing solution 2 (WS2) in sequence, with processing times of 3 min, 5 min and 5 min, respectively. Finally, the oocyte was transferred into the culture medium at 37 ${}^{\circ }$C in a humidified atmosphere of 5%${\mathrm{CO}}_{2}$.

### 2.8. Evaluation of Success Rate and Oocyte Survival Rate

An operation was considered successful when the system achieved all steps of the vitrification protocol and successfully transferred the processed oocyte to the Cryotop. Manual vitrification experiments were also performed by embryologist. The post-freezing survival rate of thawed oocytes was measured to further quantify the performance of the system. Survivability was measured directly by examining the morphology of oocytes after thawing. According to commonly used criteria to judge the survivability of oocyte cryopreservation, an oocyte was considered healthy/alive when it had no abnormal shape, membrane damage or degeneration or fragmentation of cytoplasm.

### 2.9. JC-1 Staining and ROS Level Measurement of the Oocytes

The MMP was evaluated using the JC-1 probe (Cat. T3168, Invitrogen, Waltham, MA, USA). JC-1 dye exhibits potential-dependent accumulation in mitochondria, as indicated by an emission shift in the fluorescence from green (529nm) to red (590nm). Thus, mitochondrial depolarization could be indicated by a decrease in the red/green fluorescence intensity ratio. Thawed oocytes were cultured in M2 medium containing 2 mM JC-1 for 30 min at 37 ${}^{\circ }$C, after which they were washed 3 times with PBS. The samples were then immediately imaged by confocal microscopy. To measure ROS level in thawed oocytes, carboxy-H2DCFD (Cat. C400, Invitrogen, Waltham, MA, USA), a fluorescent oxidative stress indicator, was applied in this assay. Oocytes were pretreated with M2 medium containing 10 mM $H_{2}O_{2}$ for 5 min. They were then washed and incubated with M2 medium containing 10 μM carboxy-H2DCFD for 30 min at 37 ${}^{\circ }$C, after which they were washed 3 times with PBS and then immediately imaged by confocal microscopy.

### 2.10. Confocal Imaging

Images of the fluorescently labeled oocytes were acquired using a ZEISS LSM 900 with an Airyscan 2 laser scanning confocal microscope and Hybrid Detectors (HyD). Images were captured with a HC PL APO ×20/0.7 NA CS2 dry objective lens. Alexa Fluor 488, Alexa Fluor 546 and DAPI fluorescence was captured with an argon-ion laser operating at 488nm, a HeNe laser operating at 561nm, or a diode-pumped solid-state laser operating at 405nm using 488nm excitation/519nm detection, 552 excitation/575nm detection, and 405nm excitation/461nm detection, respectively.

### 2.11. Statistical Analysis

The data were analyzed with Minitab version 18 (Minitab Inc., State College, PA, USA) using the student’s *t*-test. *p*-values less than 0.05 were considered to be statistically significant. Graphs were generated using Microsoft Excel and figures were prepared with CorelDraw version X8 (Corel Corp., Ottawa, ON, Canada).

## 3. Results and Discussion

### 3.1. Fluid Simulation and Performance Characterization of the Open Microfluidic Chip

**Figure 6:**
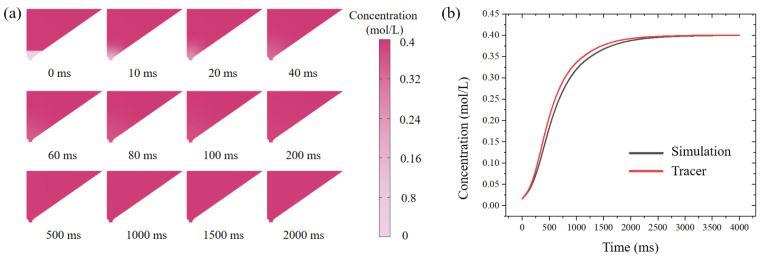
Theoretical and experimental VS concentration around the oocyte with respect to time. (**a**) Simulated time-dependent sucrose concentration in the oocyte chamber under diffusion condition (initial concentration upper: 0.4 mol/L; bottom: 0 mol/L). (**b**) Gray: the numerically predicted sucrose concentration in the oocyte chamber over time; Red: the experimentally measured intensity of the fluorescent tracer in the oocyte chamber.

The rate of change of CPAs concentration in the oocyte chamber had a significant effect on vitrification. We selected sucrose and numerically simulated its transient concentration in the oocyte chamber. Once the VS was introduced into the oocyte chamber, the sucrose concentration changed due to diffusion between the newly introduced VS and the residual ES, according to Equation (1); the snapshots of the simulated diffusion process are shown in Figure 6a. The average concentration of CPAs in the bottom region of the oocyte chamber was calculated, and its variation with respect to time is shown in Figure 6b. The sucrose concentration around the oocyte increased exponentially within 0–1000 ms and reached a plateau after 2000 ms. The constant sucrose concentration after 2000 ms indicated that the diffusion of VS was complete. Therefore, the time for VS to reach the desired concentration by diffusion was estimated to be 2 s, which was negligible compared to the processing time (30 s), validating the rationality of our designed system.

Moreover, we analyzed the diffusion of tracer in the chip and the changes in fluorescence intensity at the bottom of the oocyte chamber at different time points using image J@(NIH, USA), shown in Figure 6b. The bottom fluorescence increased exponentially within 1000 ms and reached a plateau at 2000 ms, which was consistent with the simulation results. Tracer experiments and numerical simulations showed that, on our designed open microfluidic chip, CPAs could be introduced in the form of a concentration gradient, which would reduce the risk of osmotic shock due to CPAs mutation compared with the manual Cryotop method.

**Figure 7:**
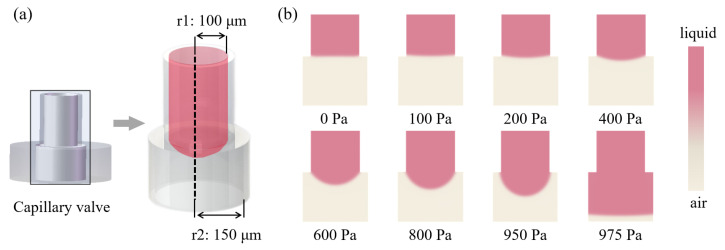
Numerical simulation of critical burst pressure of capillary valve. (**a**) The capillary valve modeling. (**b**) Deformation of the lower meniscus under different external pressures (liquid: pink; air: gray).

In order to simulate the critical burst pressure of the capillary valve, the VS with higher density and greater gravity was selected as the candidate, and the contact angle of the PMMA chip was 95${}^{\circ }$. Under different external pressures, the change of the meniscus of the capillary valve is shown in the Figure 7. Obviously, the protruding degree of the lower meniscus increased with the increase in external pressure, and the contact angle also increased. When the external pressure reached 975 Pa, the capillary valve was opened, and the liquid flowed to the outside. Therefore, 975 Pa was considered as the critical burst pressure of the capillary valve, which agreed well with the theoretical prediction (950 Pa) of Equation (3) above.

To further evaluate the reliability of the capillary valve, a constant pressure pump was used to test the critical burst pressure of the valve.The valve was opened when the pressure reached 900 Pa. The slightly lower critical pressure than the calculated values could be attributed to the output fluctuation of the pump. The hydraulic pressure of VS in the fully filled oocyte chamber was about 30 Pa, which was far less than the critical burst pressure. Therefore, we believed that the designed capillary valve could reliably block the liquid during CPAs treatment and allowed oocytes to pass through under exerted pressure.

**Figure 8:**
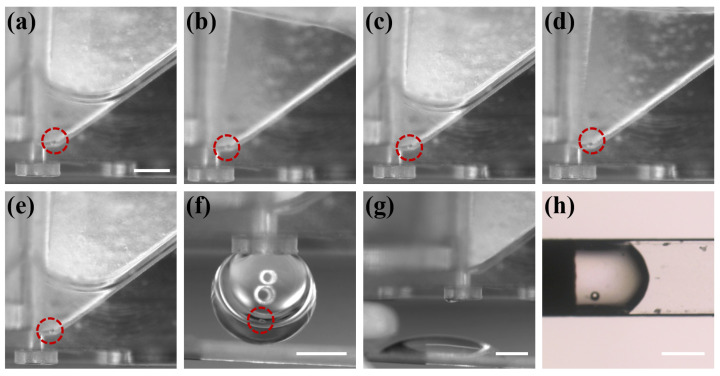
The microparticle was tracked by the imaging system over the entire process of system vitrification. (**a**) The operator placed the microparticle in the chip. (**b**–**g**) The microparticle completed vitrification pretreatment and was transferred to Cryotop. (**h**) The droplet on Cryotop contained microparticle. Scale bars in (**a**,**f**,**g**,**h**) are 400 μm.

After verifying the reliability of two key parts of the chip, the overall workflow of the chip was characterized. The PS microparticle within the chip was tracked with the imaging system and the different processing steps were recorded, as shown in Figure 8. The operator first placed the microparticle in the oocyte chamber (as shown in Figure 8a), and then performed the abovementioned (Figure 4) operation process. Steps 1–4 were the CPAs treatment process (as shown in Figure 8b–e), in which steps 2 and 4 were the removal of CPAs. The immobilization of the microparticle in the oocyte chamber indicated that the capillary gap had the effect of blocking the passage of microparticle/oocyte, and the different depths of the chambers on both sides could ensure that there was a certain amount of liquid remaining after the unloading of CPAs to avoid osmotic pressure damage caused by the rapid evaporation of the liquid. There was no leakage of fluid in the oocyte chamber prior to step 5, demonstrating the reliability of the capillary valve. When the vitrification pretreatment was completed, the operator pressed the handle to introduce external pressure. Figure 8f shows that the external pressure reached the critical burst pressure of the capillary valve, and the droplet containing the microparticle was about to be transferred to the Cryotop. Figure 8g shows the transfer of the droplet to the Cryotop with almost no liquid residue in the chip, and Figure 8h is the microparticle transferred to the Cryotop after processing. So far, the system has completed the entire vitrification pretreatment and transfer.

### 3.2. Success Rate and Oocyte Survival Rate

As summarized in Table 1 and Table 2, the system achieved a similar success rate compared with manual operation in the GV stage (100% versus 97.3%) and MII stage (100% versus 97.9%). Among the system vitrification of 45 oocytes in the GV stage and 39 oocytes in the MII stage, all of them were successfully vitrified. During manual operation, the human operator might occasionally fail to locate the oocyte within the limited time of the protocol and fail to aspirate the oocyte. The survival rates of thawed oocytes were calculated to further quantify the system performance. Compared with the manual Cryotop operation, the survival rate of the system was slightly higher (GV stage: 97.4% versus 94.4%, MII stage: 97.8% versus 97.8%), which was due to the different loading methods of CPAs and the operational reproducibility. On the one hand, the CPAs concentration changed abruptly under manual operation, while the CPAs concentration in the microfluidic chip changed more gently. On the other hand, in experiments involving complex manual operations, it was difficult for the operator to ensure the consistency of the experiment. Compared with the microfluidic system proposed by Jiang et al. [25], our system had a higher survival rate (97.8% versus 75.0%). This might be attributed to the employment of commercial Cryotop in our platform, which had a higher cooling rate than the independently designed carrier. With the precise control of volume and treatment time in CPAs, the system reduced the risk of overexposure and osmotic shock, and achieved a desirable oocyte survival rate as manual operation.

### 3.3. No Difference between Manual Manipulation and Microfluidics System for Oocytes Cryopreservation

**Figure 9:**
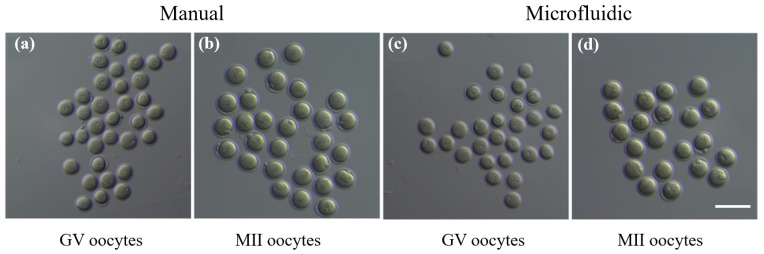
Evaluation of oocytes thawed from microfluidics vitrification via comparing with traditional manual vitrification method. (**a**,**c**) GV oocytes and (**b**,**d**) MII oocytes (with polar body extruded) were thawed from manual vitrification and microfluidics vitrification. Scale bar is 100 μm.

The GV oocytes and MII oocytes were cryopreserved via manual vitrification and microfluidics vitrification, respectively. We firstly demonstrated that there was no big difference on morphological changes between both groups of GV or MII oocytes. No obvious degeneration or fragmentation of cytoplasm was detected in thawed oocytes (Figure 9), which suggested that the oocytes could survive vitrification and thawing procedures performed either via traditional manual operations or the microfluidics system.

**Figure 10:**
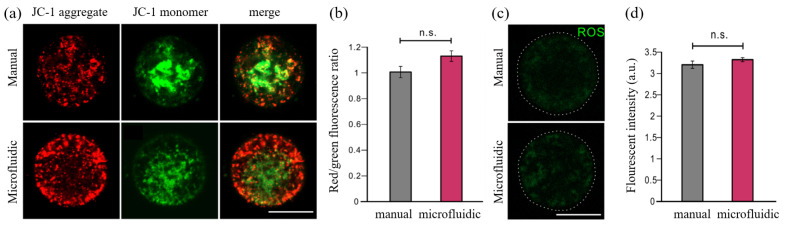
JC-1 staining and ROS level measurement of the oocytes. (**a**) The MMP level of thawed MII oocytes was measured by JC-1 staining. (**b**) The red/green fluorescence ratio of oocytes was quantified between manual and microfluidic groups. (**c**) The ROS level of thawed GV oocytes between manual and microfluidic group. (**d**) The ROS levels were measured and quantified in two groups. The students’ *t*-test was used to compare the difference in two groups. n.s. means no significant difference between two compared groups. Scale bar is 50 μm.

To further evaluate the quality of vitrification-thawed oocytes from these two methods, we next performed several biomolecular experiments to test whether the functional changes occurred in these oocytes. JC-1 live staining experiments were performed on thawed MII oocytes to check the MMP level, which is a typical biomarker to assess the physiologic function of mitochondrion in oocytes. The red fluorescent signals labeled JC-1 aggregates and green fluorescent signals labeled JC-1 monomers. The ratio of red/green fluorescence represented the level of MMP. According to this red/green fluorescence ratio, the representative images captured via a confocal microscope demonstrated a slightly better MMP level in the microfluidics group compared with that in the manual group. However, there was no significant difference after statistical analysis between both groups (Figure 10a,b).

Moreover, the ROS levels from cytoplasm of vitrification-thawed MII oocytes were measured as one of the criteria to evaluate the biological functions of these cryopreserved oocytes. The representative images showed that the thawed oocytes in both groups have a comparable ROS level after the recovery from $H_{2}O_{2}$ pretreatment. The low expression of green fluorescence revealed the normal ROS levels and healthy conditions in these thawed oocytes, whereas the high ROS levels usually represent a high-cell-stress condition or the preliminary phase of apoptosis [39]. The quantification of green fluorescence intensity demonstrated no significant difference between both groups, suggesting that the microfluidics vitrification method conducted similar results with respect to the traditional vitrification method (Figure 10c,d). Overall, based on the results collected from above biomolecular experiments, we conclude that no morphological changes or functional changes were detected from the cryopreserved oocytes via both systems.

## 4. Conclusions

In summary, we demonstrate a microfluidic platform for achieving standardized vitrification of oocytes, which is compatible with the commonly used Cryotop method. Theoretically, our platform is applicable to all embryos or oocytes following the standard vitrification process. With an open microfluidic chip, the system realized the gentle loading of CPAs, the positioning of the oocyte and the transfer of oocyte to a Cryotop. The flow behavior inside the chip, i.e., the variation of CPAs concentration around the oocyte and the reliability of the capillary valve, was systematically investigated through theoretical and experimental methods. Mouse GV and MII oocytes were then selected as biological samples to verify the vitrification effect of the open microfluidic chip platform. The results demonstrate the considerable stability and reliability of the designed chip to complete the vitrification pretreatment and transfer of the oocyte within the specified operating time, with a success rate of 100%. Evaluating the quality of thawed mouse GV/MII stage oocytes vitrified by two methods, our system showed a higher survival rate (97.6% versus 94.0%) and consistent MMP and ROS levels compared with the manual group conducted by the senior embryologist. To the best of our knowledge, this is the first report of the microfluidic system based on the Cryotop gold standard to complete the vitrification procedure without any subsequent processing, making it accessible to embryologists who are not familiar with microfluidic manipulation and also much lower in cost than commercial automated vitrification platforms (3000 USD versus 75,000 USD) [40]. The estimated cost of 3000 USD for our platform includes the production of microfluidic chips in addition to machining of mechanical structures and the affiliated sensors and actuators. Considering the tiny size and preciousness of human oocytes, the manual operation should be very precise and reliable, which improves the difficulty of training a qualified operator. Facilitated by our platform, the vitrification process is effectively simplified and standardized, which reduces the requirement for operators. It normally takes 15–20 min for a skillful human operator to manually conduct the treatment and freezing processes for an oocyte. With the help of our platform, this period can be controlled to within 15 min. It is worth noting that our platform can effectively avoid misoperation during manual operation and eliminate the differences caused by individual operators. Finally, we believe that the developed vitrification microfluidic system will greatly facilitate assisted reproduction and provide a standard solution for germ cell vitrification.

## Figures and Tables

**Table 1:** Experimental results of mouse GV stage oocytes vitrification.

	GV Stage		
	Number	Success Rate (%)	Survival Rate (%)
Manual	37	97.3	94.4
Microfluidic	39	100	97.4

**Table 2:** Experimental results of mouse MII stage oocytes vitrification.

	MII Stage		
	Number	Success Rate (%)	Survival Rate (%)
Manual	47	97.9	97.8
Microfluidic	45	100	97.8
Jiang et al. [25]	20	/	75

## Data Availability

Not applicable.
